# Letter from Dr. P. H. Strong

**Published:** 1862-12

**Authors:** P. H. Strong

**Affiliations:** Frederick City Hospital


### LETTER FROM DR. P. H. STRONG.

FREDERICK CITY HOSPITAL,
November 10, 1862.

DR. MINER, Sec'y Buffalo Medical Association:

*Dear Sir:*—I much regret my inability to be present with the Society as it meets to do honor to the memory of the lamented Wilcox. But I must not be denied the sad privilege of furnishing a few items pertaining to the last two or three months of his life, as my feeble tribute. While others will b tter descant upon his earlier history, perhaps no other member of the Society has had quite so good opportunities for observation, as regards the closing months of his life, as the undersigned. I feel his loss all the more keenly, for the two-fold reason—1st, that it was his almost affecting representation of the overwhelming duties thrown upon him by the disastrous battles and retreats in front of the Capital, indicating my name, among others, as one who ought to come to his relief—that fixed my purpose, and precipitated my departure to join the service; and 2d, his was the last face, among my old-time medical friends, that my eyes have rested on.

With your leave, I will present what I have to say of him in the form of a brief sketch of that eventful period of his life. In the early days of September, when on my way to join the army, I providentially met his associate of the 21st Regiment, my friend Dr. Peters, in Washington. He was to re-join his Regiment, then a few miles north of the City, that very day. And within a few hours after arriving in W. we were in saddle and, en route to join his command. We did not catch up with it till the next morning, and when we did, I have a very vivid remembrance of the sorrowful impression which Dr. Wilcox's look gave me. I had never seen him look so emaciated, jaded and care-worn. The army for two or three weeks preceding that date, had had an unintermitted succession of battles by day and retreats by night. The constant tension upon the energies, mental and physical, by this series of defeats, retreats, night vigils and apprehensions, combined with the deprivation of food, had wrought its work upon the army, rank and file; and upon none, that I saw, was it quite so evident as upon Dr. W. I found the Surgeons' tent, and endeavored to relieve the Doctor as much as I could, but I had to insist upon it very urgently, before he would consent to be relieved to any appreciable extent.

It will be remembered that the object of the campaign, then in progress, was the annihilation or the expulsion of the rebel hordes from this State, (Md.) and our route lay generally toward this City in western Maryland. For four or five days nothing eventful occurred. The weather was fine; the army marched very leisurely; food was abundant and eaten regularly; sleeping in camp was delicious, and all were exhilarated with hope, begotten by *McClellan's* newly assuming command. Methought those few days, with perhaps some relief from duty, wrought an agreeable change in the Doctor's appearance. His old cheerfulness and powers of joking and raillery seemed to return, and it seemed quite like old times to be with him. Five days' march brought us to within five miles of this city, and to Saturday night. Sunday morning (14th Sept.) we were aroused at 3 o'clock, and on the march by earliest light. Our route lay through this city westward; and very early in the morning the booming artillery, estimated to be fifteen or twenty miles distant, told us that there was work at hand. Eight miles west of this, a little after noon, we came to McClellan's head quarters, which were some three miles east of the South Mountain range, where the battle was even then in progress.

I had stipulated at the Surgeon-General to serve in the field. But on arriving at head quarters the Medical Director, in view of the results from the coming conflicts, said I would much oblige him by reporting myself back to this city for hospital duty. It was a sore disappointment to me, but its reasonableness was apparent, and I consented to give up my long cherished wish to aid Dr. Wilcox, and to go to the hospital. The Doctor saw my disappointment, but said "*I should see and assist in one battle, any how.*" He very kindly agreed to ride with me ahead of our corps, (Hooker's,) to where the artillery duel was then in full blast. In two or three hours our corps came up and took its position in line of battle, and when near sundown, it had reached the woods some three-fourths of a mile up the mountain side, (under cover of which the rebels were waiting to receive us,) Dr. Wilcox proposed to me to ride up on the battle-field to superintend the removal of our brave fellows as they fell. We did so. I confess to being satisfied with a proximity of some twenty-five rods to our line of battle. when the rattle of the musketry and the whistling of the minnies was quite terrific enough for my civic senses. But not so with the Doctor, who rode clear up to the line, dismounted and stood there, and walked about as unconcernedly as if it were a sham-fight, at a military training. I called to him not to expose himself so recklessly, and as it seemed so needlessly; but it was of no avail. He remained there some half an hour, and until the firing had slackened somewhat just there, when he as deliberately walked down, leading his horse. I chided him somewhat for his presumption, whilst I could not but admire his imperturbable coolness and bravery. Upon his giving direction to the stretcher-bearers, we went down some one-fourth of a mile to a farm house, where our wounded began to accumulate in great numbers. Four or five of us were engaged there till near midnight, when we made our way through pitchy darkness down to our ambulance, and got a few hours of sleep. At next dawn the Doctor again very obligingly offered to accompany me on to the top of the mountain, to give me a sight of a battle-field after a conflict and victory, and before its awful products were removed or interred. Soon thereafter, that forenoon, the army moved on to the still sterner fight of Antietam, on the next Wednesday, and I reported myself back to this city. That morning was the last time I saw Dr. Wilcox.

I learn from others of his Regiment that after that sanguinary battle he had duty enough put on him for *three* men, and that for a time, he bravely performed it. In his worn-out condition, with the continuous breathing of fœtid air incident to the circumstances, and with his disposition never to spare himself, of course the result could be easily foreseen. He must soon give up, or soon give out. Any one who knew the man could predicate that he would never surrender the post of duty except under the direst necessity, and then, all too late to save himself. Alas! alas! so it has proved.

I have little time, and less ability, to give a true portraiture of Dr. Wilcox. I am not sure that I can properly express my own estimate of him. What strikes me as a prominent characteristic was his whole-souled *manliness*. He had his peculiarities; nay, his foibles; we all have such, or similar. But one could count on him *sure*, in a trying pinch, such as is apt to test friendship. Destitute of many early advantages, he accomplished much of self-culture, and was a physician of rare good judgment. He was a man with whom, though it was pleasant to agree, it was almost as agreeable to differ. He could *take* as well as give strictures and jokes, (so-called,) a quality none too common. Though in some respects a *rough* diamond, he was a diamond of rare excellence, nevertheless. We shall miss him in the profession, the interests of which he was ever ready to serve, and the honor and advancement of which he ever strove to promote. Our Association will miss him as one of its earliest and most constant friends. Overlooked though it be, Buffalo General Hospital is more indebted to him for its first breath of life, than to any other one man.

But to return to his more recent life. I speak from the right standpoint, and of what I do know when I say that since he joined the army he has done honor to himself, and honor to our noble profession. *No* surgeon has labored *more* indefatigably and successfully to promote the comfort and health of the army than he, and I believe *few* have *done so much.* But enough—alas! he has gone from us forever. Let us lay the lesson of the brevity and utter uncertainty of life to heart—cherish his memory—and strive to emulate his many excellent traits, and to imitate his many good works.

Respectfully,

P. H. STRONG.

